# Supplementary material for: Distributing task-related neural activity across a cortical network through task-independent connections
Source: Nat Commun. 2023 May 18;14:2851. doi: 10.1038/s41467-023-38529-y (PMC10195842; doi:10.1038/s41467-023-38529-y)
Supplement: Supplementary file 1 — Supplementary Information [file 41467_2023_38529_MOESM1_ESM.pdf]

Supplementary Material for

Distributing task-related neural activity across a cortical network through task-independent connections

Christopher M. Kim*, Arseny Finkelstein, Carson C. Chow, Karel Svoboda, Ran Darshan*

Correspondence*: chrismkkim@gmail.com, darshanr@hhmi.org

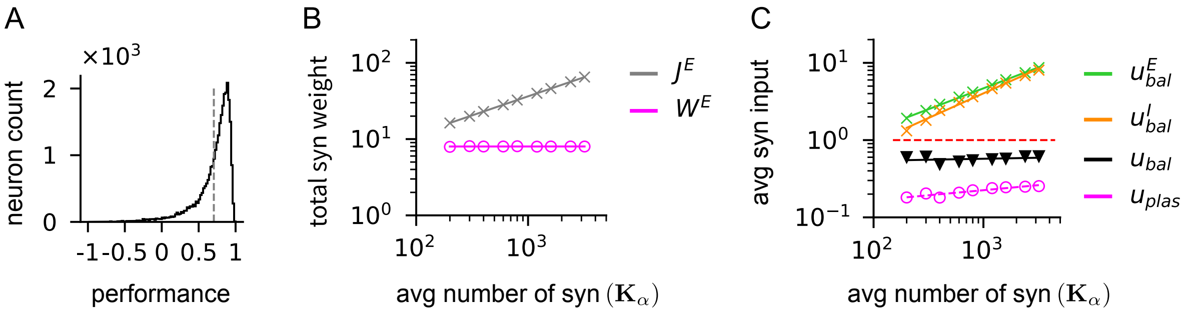

Fig. S1: Training is robust to scaling of synaptic weights and inputs. All neurons in a network consisting of $N = 30,000$ excitatory and inhibitory neurons were trained to learn sine functions with random phases. The average number of static (plastic) synapses to a neuron from population $\alpha = E, I$ in the initial network was K_α ($L_\alpha = c\sqrt{K_\alpha}$ with $c = 2$). Here, $K_E = K_I$ and the total number of static synapses per neuron $K = K_E + K_I$. The average number of static and plastic synapses was varied over a wide range to demonstrate that the training scheme was robust to increased density of synaptic connections. **(A)** Typical performance of neurons in a trained network. Average performance is shown in dashed line. Here, the performance was quantified as the correlation between the target patterns and the synaptic input to a neuron that learned the target. **(B)** The strength of both static and plastic synapses was proportional to $1/\sqrt{K_\alpha}$. Therefore, the total weight of plastic synapses to a neuron remained constant in initial networks, independently of K_α (W^E: total weight of plastic excitatory synapses to neurons). In contrast, the total weight of static synapses to a neuron increased proportionally to $\sqrt{K_\alpha}$ [44] (J^E: average weight of static excitatory synapses to neurons). **(C)** Strength of different types of synaptic inputs to neurons in networks trained over a wide range of K_α. For each K_α, networks were trained until the average performance reached 0.6. Trained networks showed that learning was successful even as the excitatory (u^E_{bal}) and inhibitory (u^I_{bal}) balanced inputs coming through the static synapses increased with $\sqrt{K_\alpha}$, while the plastic inputs (u_{plas}) coming through the plastic synapses and total balanced inputs ($u_{bal} = u^E_{bal} - u^I_{bal}$) remained constant, on the order of spike-threshold (dashed red line), independently of K_α.

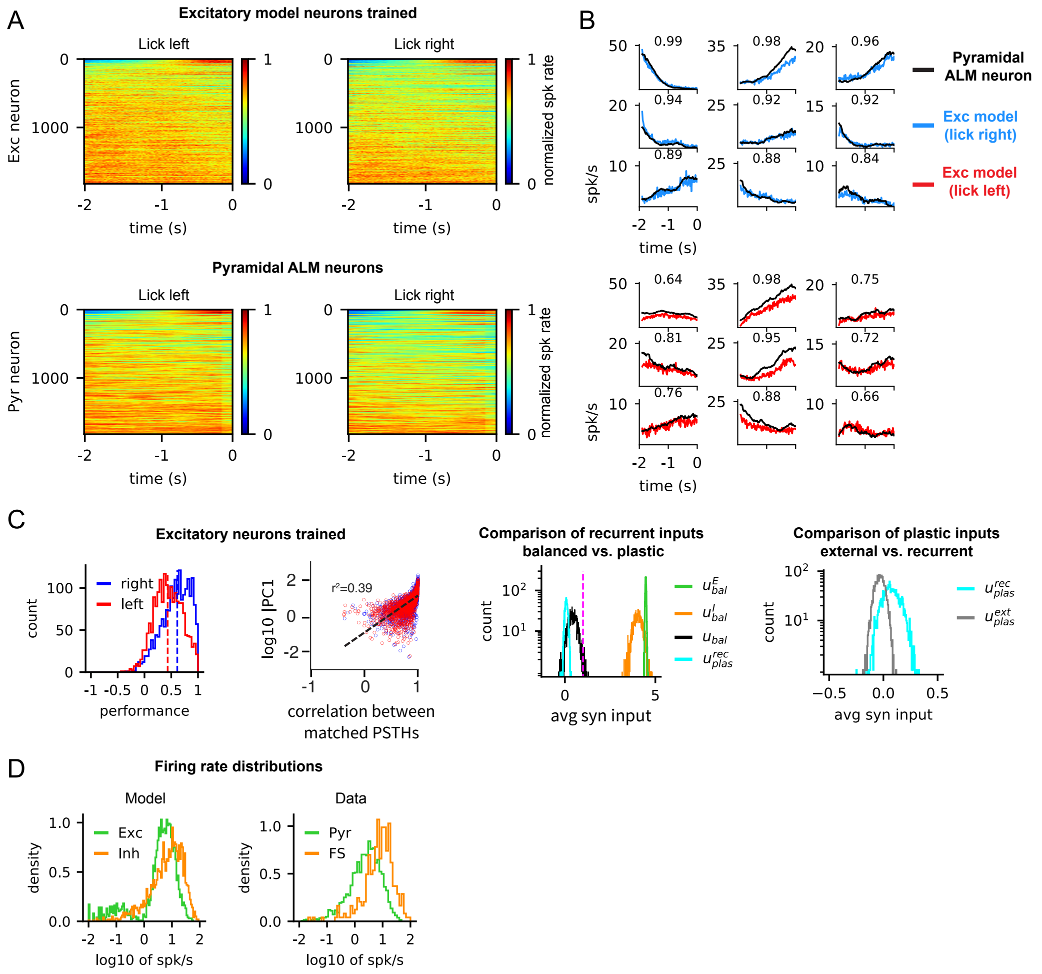

Fig. S2: Spiking activity of excitatory neurons trained to reproduce the PSTHs of pyramidal ALM neurons. The network in this figure is the same as the one presented in Fig. 2. **(A)** Normalized PSTHs of trained excitatory neurons (top) and the normalized PSTHs of pyramidal ALM neurons (bottom) that were used to train the excitatory neurons. Neurons were sorted in decreasing order according to their performance in the lick right trials. **(B)** PSTHs of nine trained excitatory and pyramidal ALM neurons with high performance on lick-right trials. PSTHs were compared for the lick-right (top) and lick-left (bottom) trials. Panels in the same position represent the activity of same neurons during the lick-right and lick-left trials. The correlation between two PSTHs are shown on each panel. **(C)** Performance (left) of all the trained excitatory neurons (average performance in dashed lines), quantified as the correlation between the PSTHs of the trained neuron and target ALM neuron. The projection of the PSTH of a pyramidal ALM neuron onto the first PC was a good indicator for how well a trained excitatory neuron could fit the pyramidal ALM neurons. Note the higher performance for lick right trials, consistent with the fact that neuronal activities were more modulated for lick right trials (Fig. S6C). This was potentially a result of asymmetries in the task design, where layer 4 neurons in the barrel cortex were photostimulated only for lick right trials. Distribution of average recurrent inputs (middle) to trained excitatory neurons, comparing balanced and plastic recurrent inputs. Here, u^E_{bal}, u^I_{bal} are the excitatory and inhibitory balanced inputs, respectively, to the trained neurons through the static synapses. u_{bal} is the sum of u^E_{bal} and u^I_{bal}. u_{plas} is the plastic input to the trained neurons through the plastic synapses. Distribution of average plastic inputs (right) to trained excitatory neurons, comparing external and recurrent plastic inputs. **(D)** Distribution of the firing rates of neurons in a trained network (left) and ALM neurons (right).

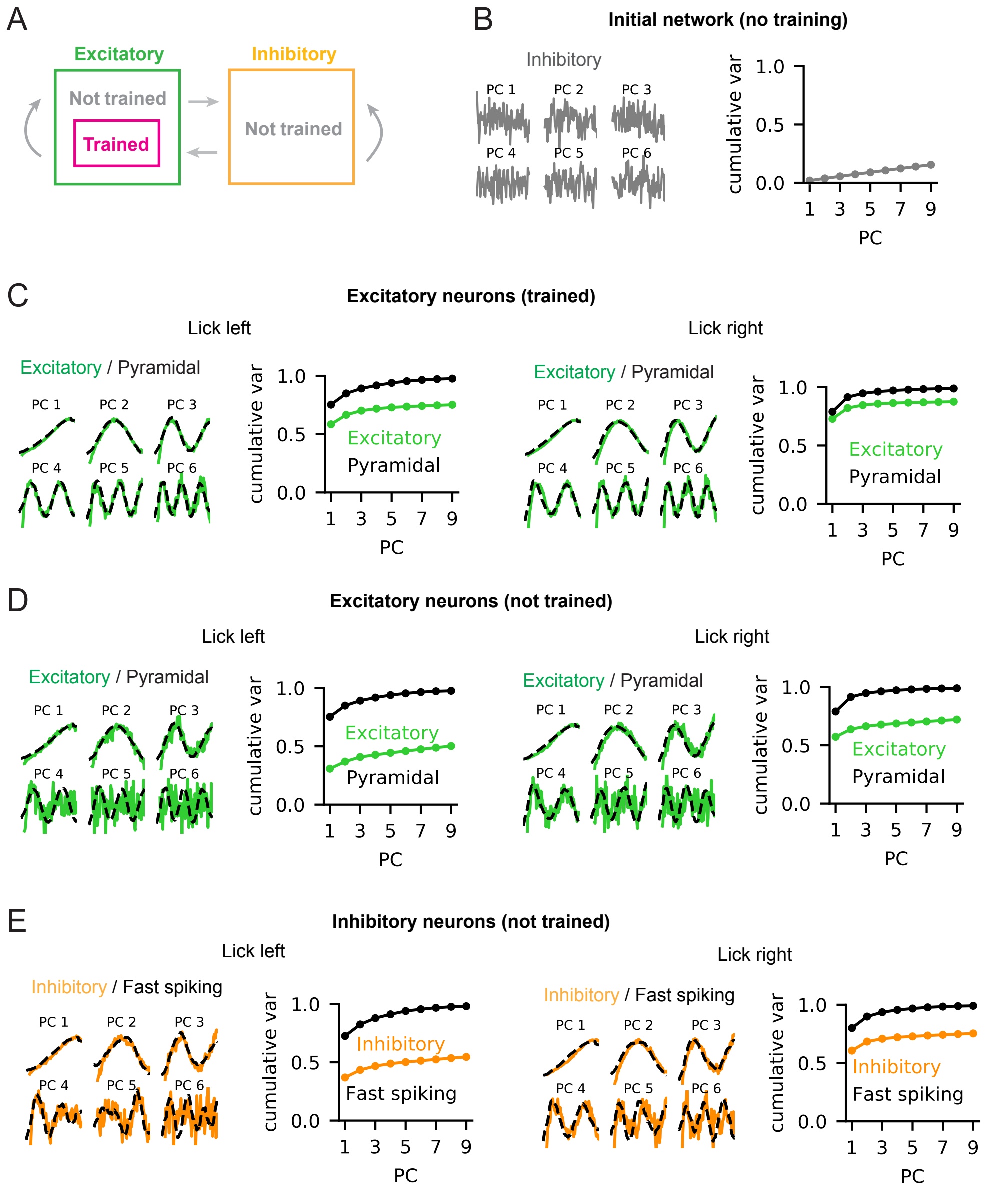

Fig. S3: PCA on the PSTHs of the initial network, trained excitatory neurons, untrained excitatory neurons and untrained inhibitory neurons. **(A)** Schematic of the Subset Training, where only 1824 excitatory neurons out of total 2500 excitatory neurons were trained. It is the same trained network presented in Fig. 2. **(B)** The first six PCs of PSTHs of the inhibitory neurons in the initial network before training (left). The cumulative variance explained by the PCs (right). **(C)** PCA on the PSTHs of the trained excitatory model neurons and pyramidal ALM neurons for the lick-left and lick-right trial types. **(D)** PCA on the PSTHs of the untrained excitatory model neurons and pyramidal ALM neurons for the lick-left and lick-right trial types. **(E)** PCA on the PSTHs of the untrained inhibitory model neurons and fast-spiking ALM neurons for the lick-left and lick-right trial types.

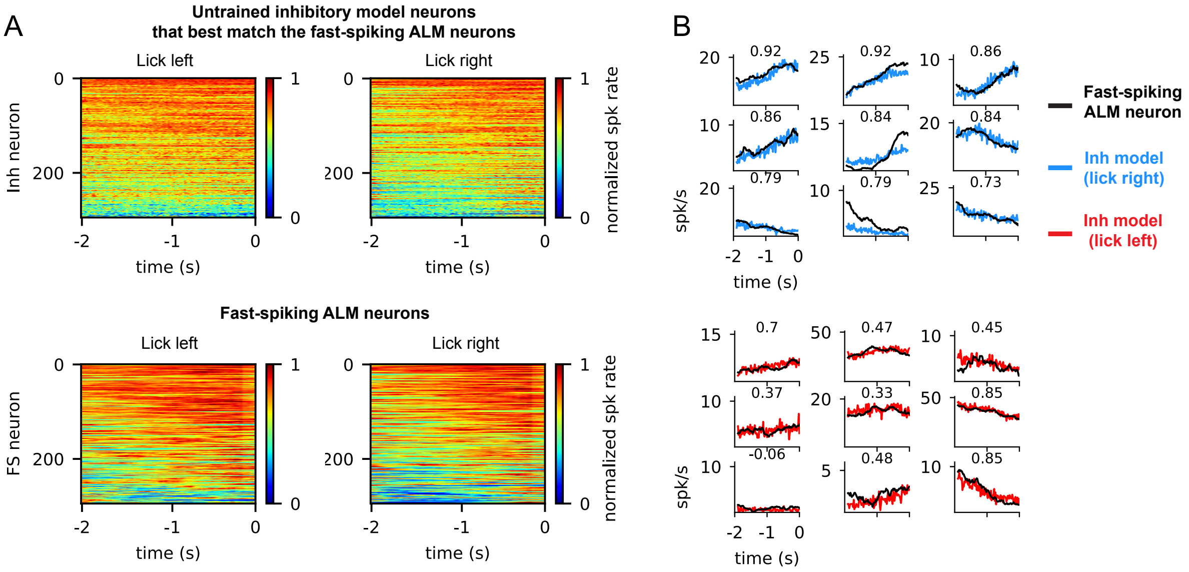

Fig. S4: Spiking activity of untrained inhibitory neurons that best match the spiking activity of fast-spiking ALM neurons. The network in this figure is the same as the one presented in Fig. 2. **(A)** Normalized PSTHs of the untrained inhibitory neurons (top) selected from the inhibitory population to match the PSTHs of fast-spiking ALM neurons (bottom). Neurons were sorted in decreasing order according to the goodness-of-fit for the lick-right trial. **(B)** Example PSTHs of untrained inhibitory neurons and the matched fast-spiking ALM neurons. Panels in the same position represent the activity of same neurons during the lick-right (top) and lick-left (bottom) trials. The correlation between the matched PSTHs are shown on each panel.

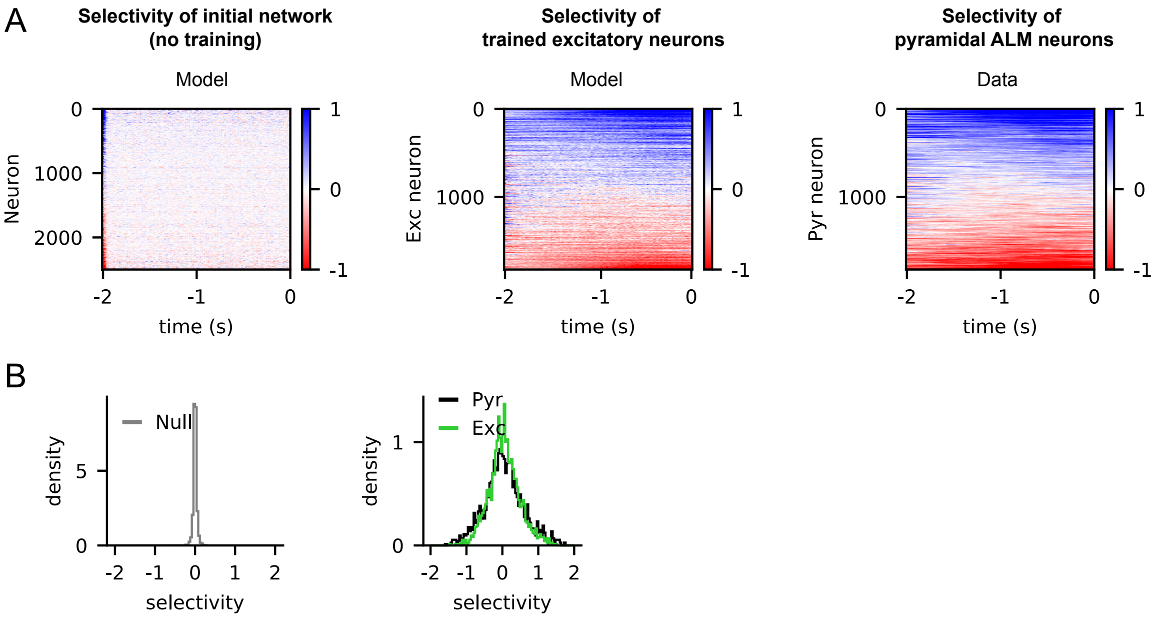

Fig. S5: Selectivity of null network neurons, trained excitatory neurons and pyramidal ALM neurons. The network in this figure is the same as the one presented in Fig. 2. The null network is the initial balanced network with no training. **(A)** Selectivity of excitatory neurons in the null network (left). Selectivity of excitatory neurons trained to generate the activity of pyramidal ALM neurons (middle). Selectivity of pyramidal ALM neurons (right). **(B)** Distributions of neurons' choice selectivity in the null network (left), the trained excitatory model neurons and pyramidal ALM neurons (right).

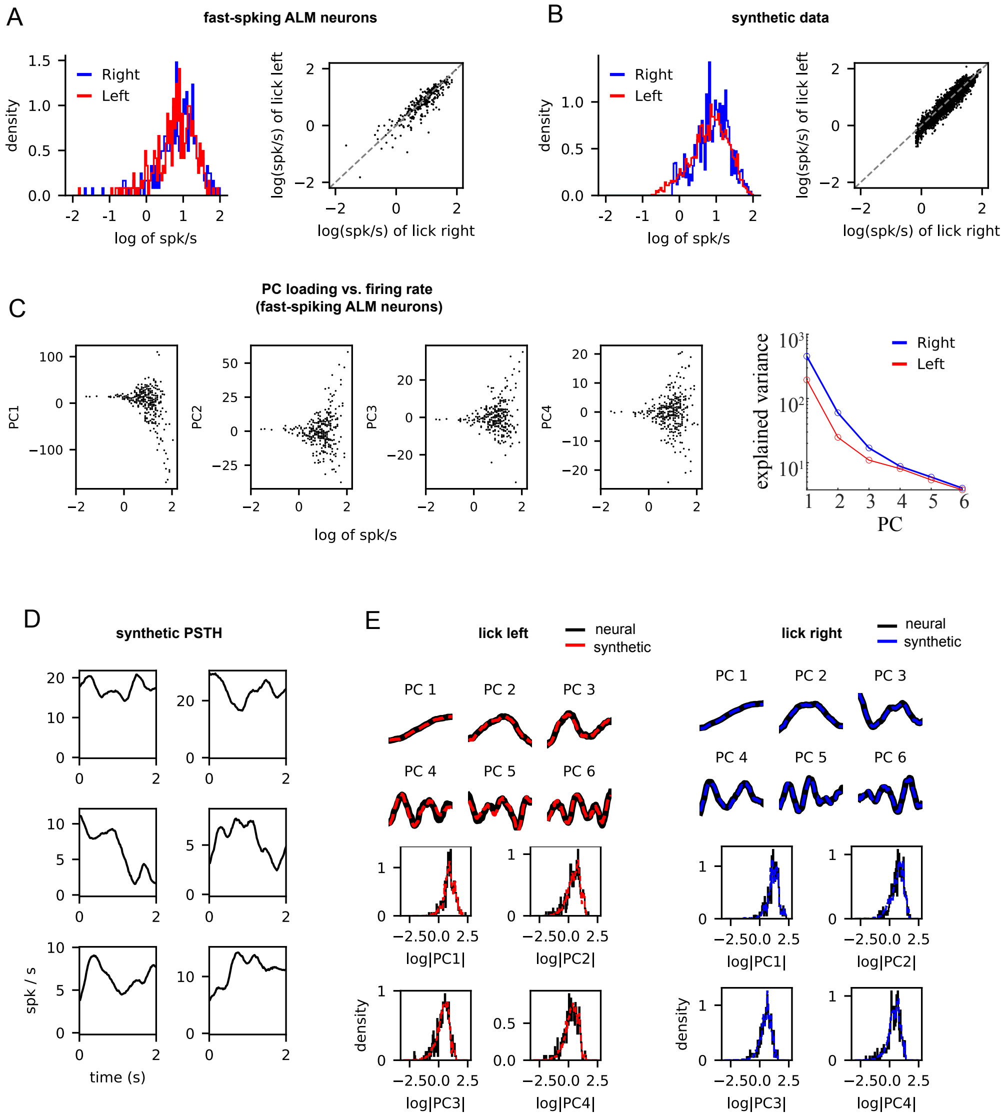

Fig. S6: Constructing synthetic neural data. **(A)** Firing rate distribution of fast-spiking ALM neurons for the lick-left and lick-right trials (left). Comparison of neuron's firing rate for two conditions (right). **(B)** Firing rate distribution of synthetic neurons. The firing rate of a synthetic neuron for the lick-right trial is sampled from ALM neuron's empirical rate distribution shown in (A, left). To get the firing rate of lick left-trials that has choice selectivity similar to (A, right), we selected fast-spiking ALM neurons whose lick-right firing rate was close to the sampled rate, and added a noise term with mean 0 and variance identical to the empirical variance of chosen ALM neuron's lick left rates. **(C)** PC loading vs. firing rate of fast-spiking ALM neurons for the lick-right trials (left). Based on the sampled rates from (B), the PC loadings to each synthetic neuron were bootstrapped from the empirical distribution of fast-spiking ALM neuron's PC loadings. Explained variance against the PC number for the right and left trials (right). Higher explained variance for right trials is presumably a result of asymmetry in task design. **(D)** Synthetic PSTHs were constructed by $\mathbf{r}_{synth} = r_0 + \sum_{n=1}^{6} c_n^{synth} \mathbf{v}_n$ where r_0 is the baseline firing rate from (B), c_n^{synth} is the nth PC's loading from (C) and $\mathbf{v}_n$ is the nth PC of fast-spiking ALM neurons. **(E)** PCA on the PSTHs of fast-spiking ALM neurons and fast-spiking synthetic neurons. The first six PCs (top) and the distributions of PC loadings (bottom) were identical. See also methods for detailed explanation.

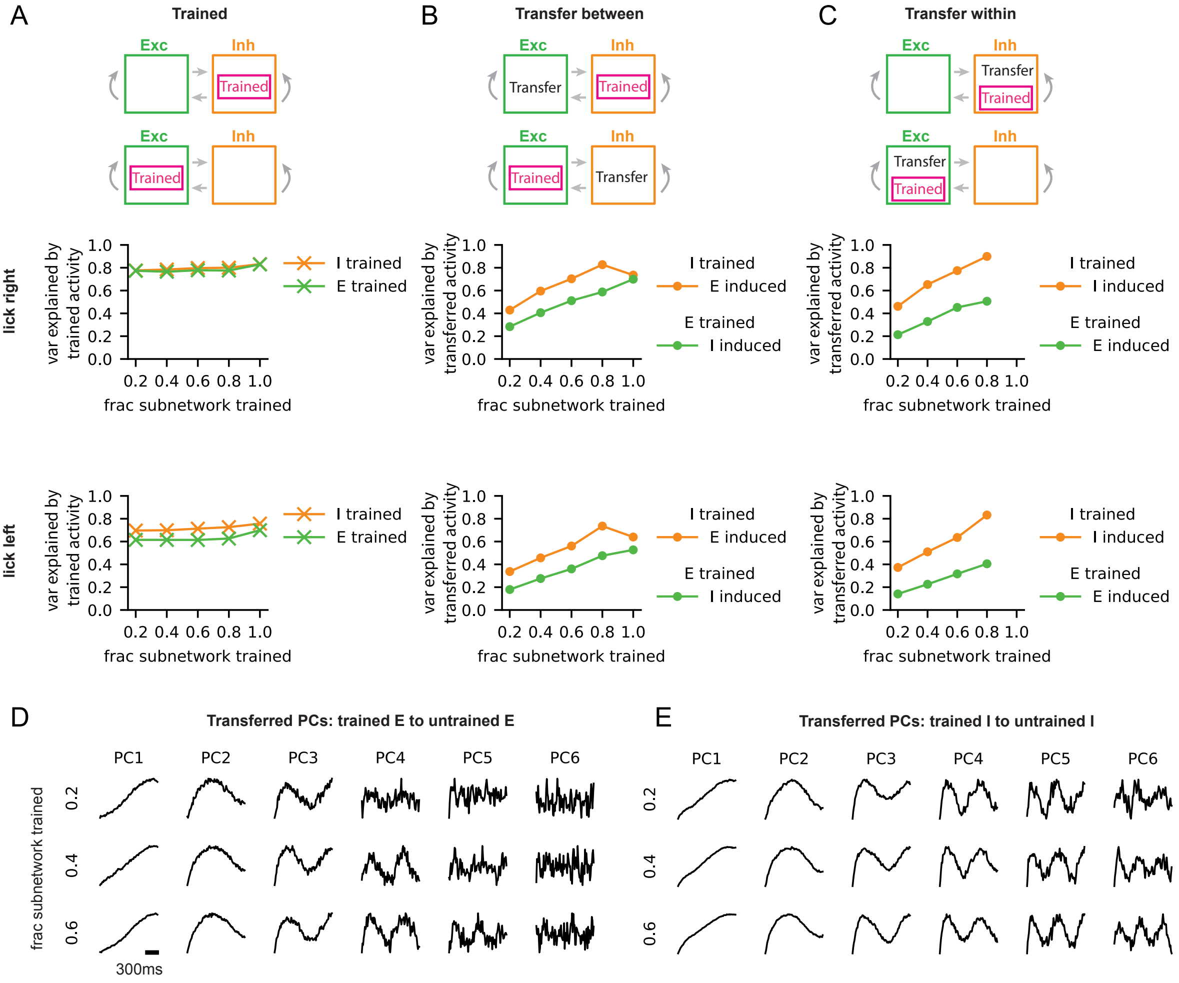

Fig. S7: Spreading of trained synthetic neural activity. Extended results from the network trained on synthetic neural data in Fig. 4. A network of size $N = 30000$ was trained using the synthetic neural data. The fraction of trained neurons within the subnetwork was varied, where the fraction equals to 1 if all the neurons within the (excitatory or inhibitory) subnetwork are trained. We considered two training scenarios (top row) where either the excitatory or the inhibitory neurons, but not both, were trained. Explained variance refers to the variance explained by the first six PCs of the PSTHs of neurons that were either trained (panel (A)) or not trained (panels (B) and (C)). **(A)** Variance explained by the subsets of trained neurons in two training scenarios for the lick-right (middle) and lick-left (bottom) trial types. **(B)** Variance explained by the untrained neurons within the subnetwork that was not trained. Two training scenarios for the lick-right (middle) and lick-left (bottom) trial types are shown. **(C)** Variance explained by the untrained neurons within the trained subnetwork. **(D)** PCs of the PSTHs transferred from the trained excitatory neurons within the trained excitatory subnetwork to the untrained excitatory neurons within the same trained excitatory subnetwork. The fraction of trained neurons was varied. This corresponds to the scenarios shown in (C). **(E)** Same as in (D), but now considered the PCs of the PSTHs transferred from the trained inhibitory neurons within the trained inhibitory subnetwork to the untrained inhibitory neurons within the same trained inhibitory subnetwork.

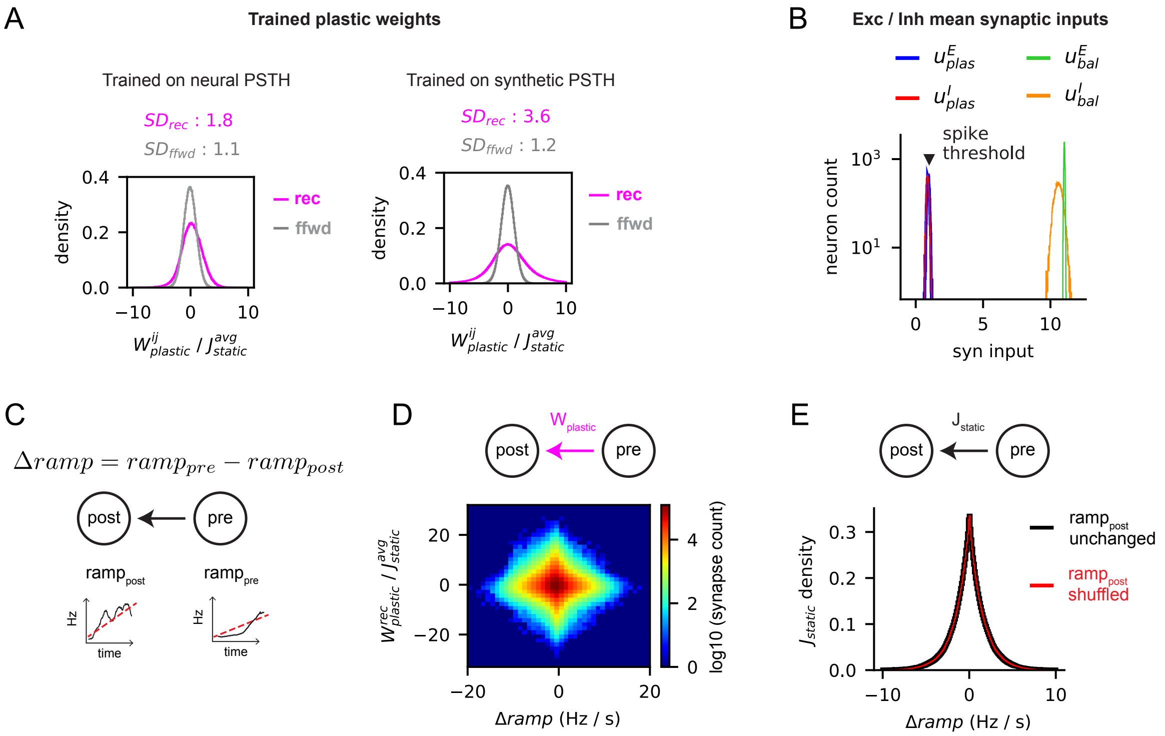

Fig. S8: Trained plastic weights are similarly strong as the static weights and correlated to ramping activity. **(A)** Trained plastic weights (recurrent and feedforward), normalized by the average static weights. Left: trained network in Fig. 2 where the 1824 excitatory neurons (out of $N_E = 2500$) were trained on neural PSTHs. Right: trained network in Fig. 4 where 40% of excitatory neurons (out of $N_E = 15000$) were trained on synthetic PSTH. Dale's principle was not respected by the plastic weights in both trained networks. SD shows the standard deviation of the normalized plastic weight distributions. **(B)** Temporal average of excitatory and inhibitory synaptic inputs to trained neurons from the network shown in (A, right). Even though the plastic weights were moderately strong, the excitatory-inhibitory plastic inputs were around spike-threshold and significantly weaker than the balanced inputs. Since the plastic weights did not respect the Dale's principle, synaptic inputs to a neuron through the positive and negative plastic weights are defined as u^E_{plas} and u^I_{plas}, respectively. **(C)** Schematic showing that $ramp_{pre}$ and $ramp_{post}$ are the slopes of the linear fit on pre- and post-synaptic neuron's PSTH, respectively. $\Delta ramp$ is the difference of the slopes. **(D)** Correlated structure between $\Delta ramp$ (i.e., difference between the ramping rates of pre- and post-synaptic neurons) and the normalized $W^{rec}_{plastic}$ (i.e., strength of the plastic synapse connecting two neurons). Strong plastic weights around $\Delta ramp = 0$ gradually decreased as the absolute value of $\Delta ramp$ increases. **(E)** Unlike the plastic weights shown in (D), the static weights only depended on four connection types (i.e. $E \to E, E \to I, I \to E, I \to I$). We tested if the ramping activity in untrained neurons might have emerged from preferential inputs from presynaptic neurons sharing similar ramping activity. We evaluated $\Delta ramp$ between untrained postsynaptic neurons and their presynaptic neurons. The density of static synapses as a function of $\Delta ramp$ is shown in black. Next, $ramp_{post}$ was randomly shuffled and $\Delta ramp$ was re-evaluated. The density of static synapses as a function of the shuffled $\Delta ramp$ is shown in red. Two distributions were identical, showing the absence of strong correlation between the ramping activity of neurons connected by static synapses.

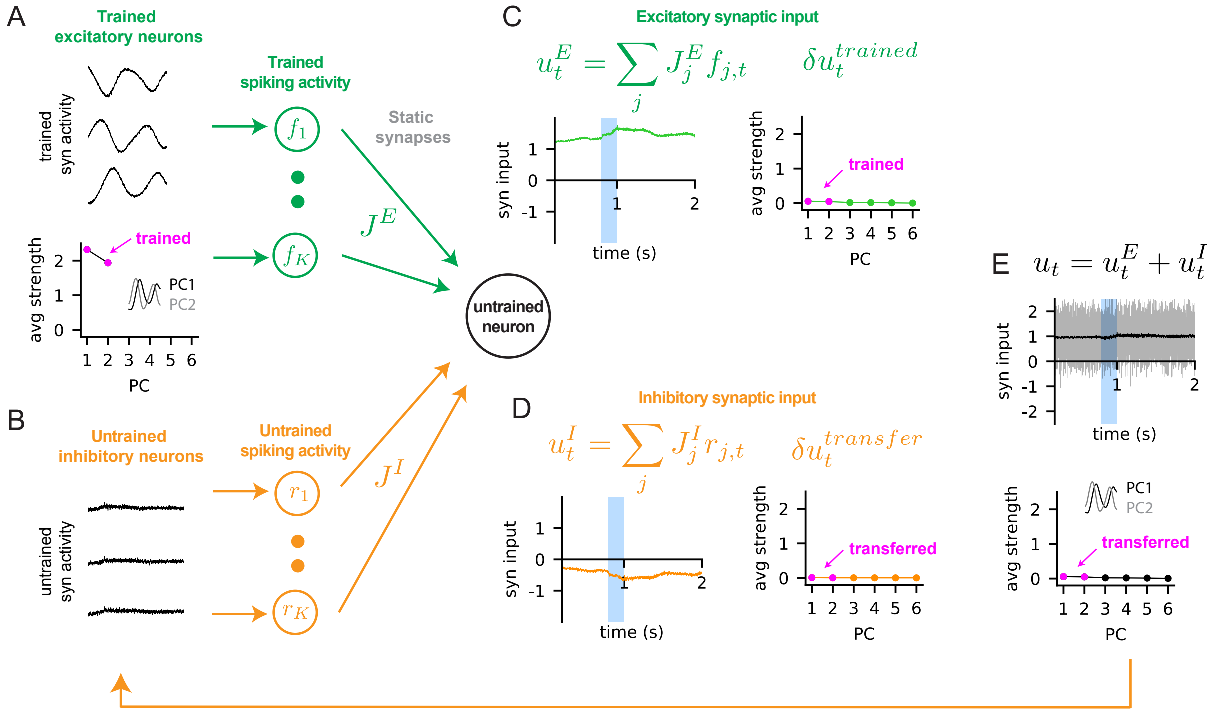

Fig. S9: Trained neural activity fails to spread to untrained neurons if the static synapses are weak. To create a weakly connected network, the weights of static synapses were scaled by $\frac{1}{K}$, instead of $\frac{1}{\sqrt{K}}$. The external inputs were adjusted to be around the spike-threshold such that the excitatory and inhibitory firing rates were around 5Hz and 12Hz, respectively, before training. Gaussian noise with mean 0 and standard deviation 0.5 was injected to the membrane equation to emulate the noisy spiking activity of the balanced network. All the excitatory neurons were trained to learn sine waves with frequency 2Hz and random phases, but the inhibitory neurons were not trained. **(A)** The synaptic activities of trained excitatory neurons followed the target sine waves (top). The PCs of the trained excitatory neurons' synaptic activity (bottom). The Fourier modes of sine waves are highlighted (magenta) and the corresponding PCs (i.e., PC1, PC2) are shown. The loading of each PC on synaptic activity was averaged over all excitatory neurons to obtain the average strength of the PCs. The first two PCs explained close to 99% of the variance. **(B)** Synaptic activity of untrained inhibitory neurons had no temporal structure. **(C)** Aggregate excitatory synaptic input to an untrained inhibitory neuron (left, u_t^E). The PCs of the temporal modulation of excitatory input (right, $\delta u_t^{trained}$) with the corresponding PCs in panel (A) highlighted (magenta). The strength of the trained PCs (i.e., PC1 and PC2) was weak. **(D)** Same as in (C), but the aggregate untrained inhibitory input to the same untrained inhibitory neuron is shown. **(E)** The total synaptic input to the untrained inhibitory neuron (top) with the Gaussian noise shown in the background (gray). The PCs of the total input to untrained inhibitory neurons (bottom) with the transferred PCs highlighted (magenta). Again, the strength of the transferred PCs was weak.

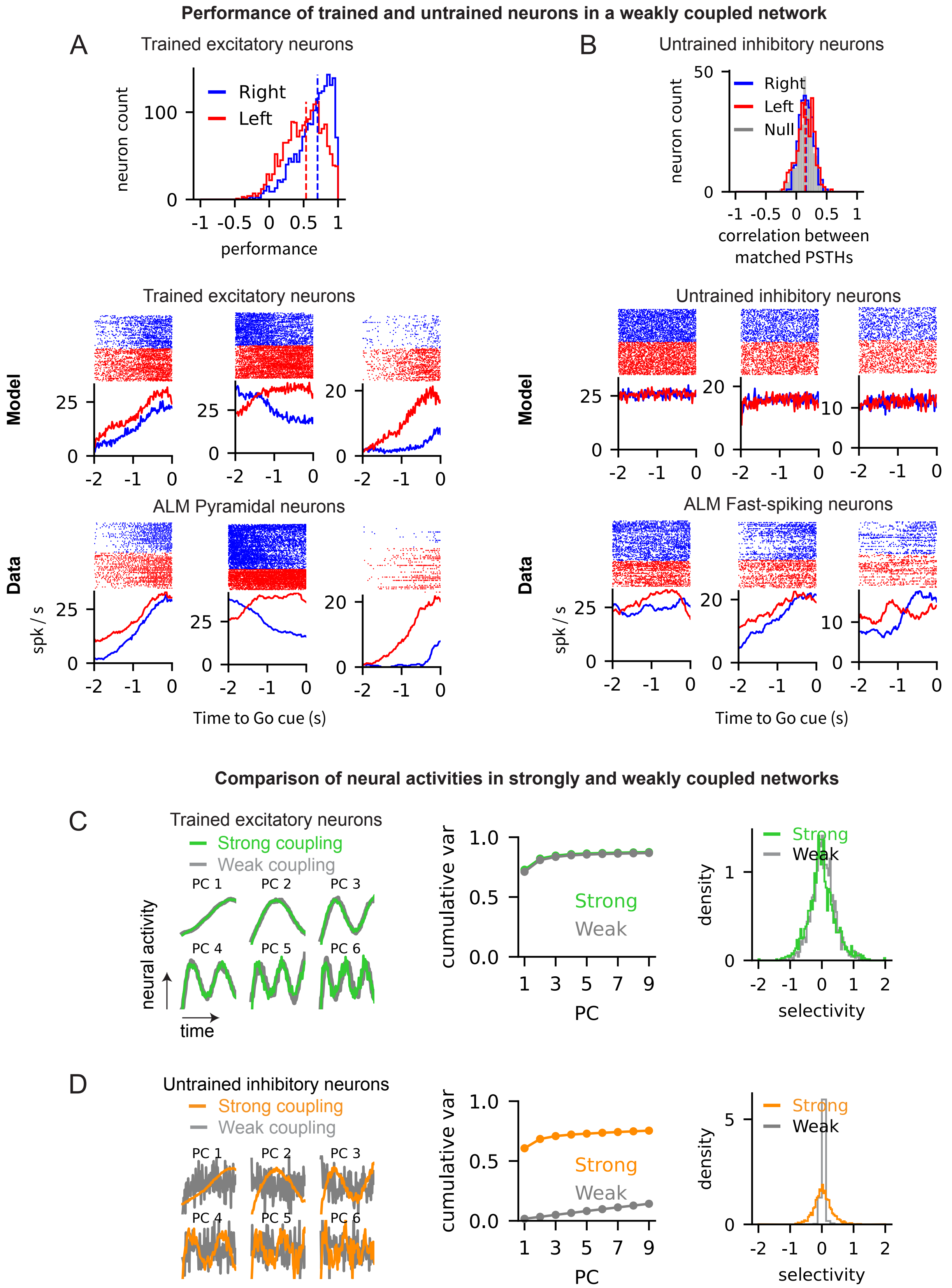

Performance of trained and untrained neurons in a weakly coupled network
A
Trained excitatory neurons
neuron count
Right
Left
performance
B
Untrained inhibitory neurons
neuron count
Right
Left
Null
correlation between
matched PSTHs
Trained excitatory neurons
Untrained inhibitory neurons
Model
ALM Pyramidal neurons
ALM Fast-spiking neurons
Data
spk / s
Time to Go cue (s)
Comparison of neural activities in strongly and weakly coupled networks
C
Trained excitatory neurons
Strong coupling
Weak coupling
PC 1
PC 2
PC 3
PC 4
PC 5
PC 6
neural activity
time
cumulative var
Strong
Weak
PC
density
selectivity
D
Untrained inhibitory neurons
Strong coupling
Weak coupling

Fig. S10: Trained ALM activity fails to spread to untrained neurons if the static synapses are weak. See Table 3 for simulation parameters. Similarly to Fig. S9, to create a weakly connected network, the weights of static synapses (denoted by J^{weak} in Table 3) were scaled by $\frac{1}{K}$, instead of $\frac{1}{\sqrt{K}}$. There were two types of external inputs: X^{weak} and $X^{gaussian}$. The baseline external inputs to excitatory and inhibitory neurons (denoted by X_E^{weak} and X_I^{weak}, respectively, in Table 3) were adjusted to be smaller than the spike-threshold such that the excitatory and inhibitory population rates were around 5Hz and 12Hz, respectively. For training, we paired each ALM pyramidal neuron ($N_{pyr} = 1824$) with a to-be-trained model excitatory neuron that had similar firing rate as the ALM neuron. However, unlike the balanced network, the firing rate distribution of the weakly coupled network was not log-normal, making it difficult to match with the log-normal firing rate distribution of ALM neurons. Therefore, to each neuron i, we injected additional constant input $X_i^{gaussian}$, which was identical to the mean synaptic input received by neuron i in an untrained balanced network of Fig. 2. Since $\{X_i^{gaussian}\}_{i=1,\dots,N}$ followed Gaussian distribution, it allowed the weakly coupled network to convert normally distributed synaptic inputs to log-normally distributed firing rates through the exponentially expanding f-I curve. The noise injected to the membrane equation for emulating the noisy spikes of the balanced network produced the exponential f-I curve. We note that neurons in a network trained without $X^{gaussian}$ captured the overall firing rate patterns of ALM neurons, but the mean firing rates of trained neurons deviated significantly from the target ALM neurons. **(A)** Performance of trained excitatory neurons (top), measured by the correlation between the PSTHs of ALM pyramidal neurons and model excitatory neuron, for the lick right and lick left trial-types. Spike trains and PSTHs of example trained excitatory neurons (middle) and ALM pyramidal neurons used for training the model excitatory neurons (bottom). **(B)** Performance of untrained inhibitory neurons (top), measured by the correlation between the PSTHs of ALM fast-spiking neurons and model inhibitory neurons that best resemble the ALM fast-spiking neurons. Null is the performance of an initial balanced network with no training, as shown in Fig. 3B. Example untrained inhibitory neurons (middle) and best-matching ALM fast-spiking neurons (bottom) are shown. **(C-D)** Comparison of population activities of trained excitatory neurons and untrained inhibitory neurons in strongly and weakly coupled networks. Here, weakly coupled network refers to the network setup discussed above, and strongly coupled network is identical to the trained balanced network in Fig. 2. (C) The principal components (PCs) of the PSTHs of trained excitatory neurons in the strongly and weakly networks (left). Cumulative variance of the PCs in both networks (middle). The choice selectivity of trained excitatory neurons in both networks (right). (D) Same as in (C) but for the untrained inhibitory neurons in strongly and weakly coupled networks.

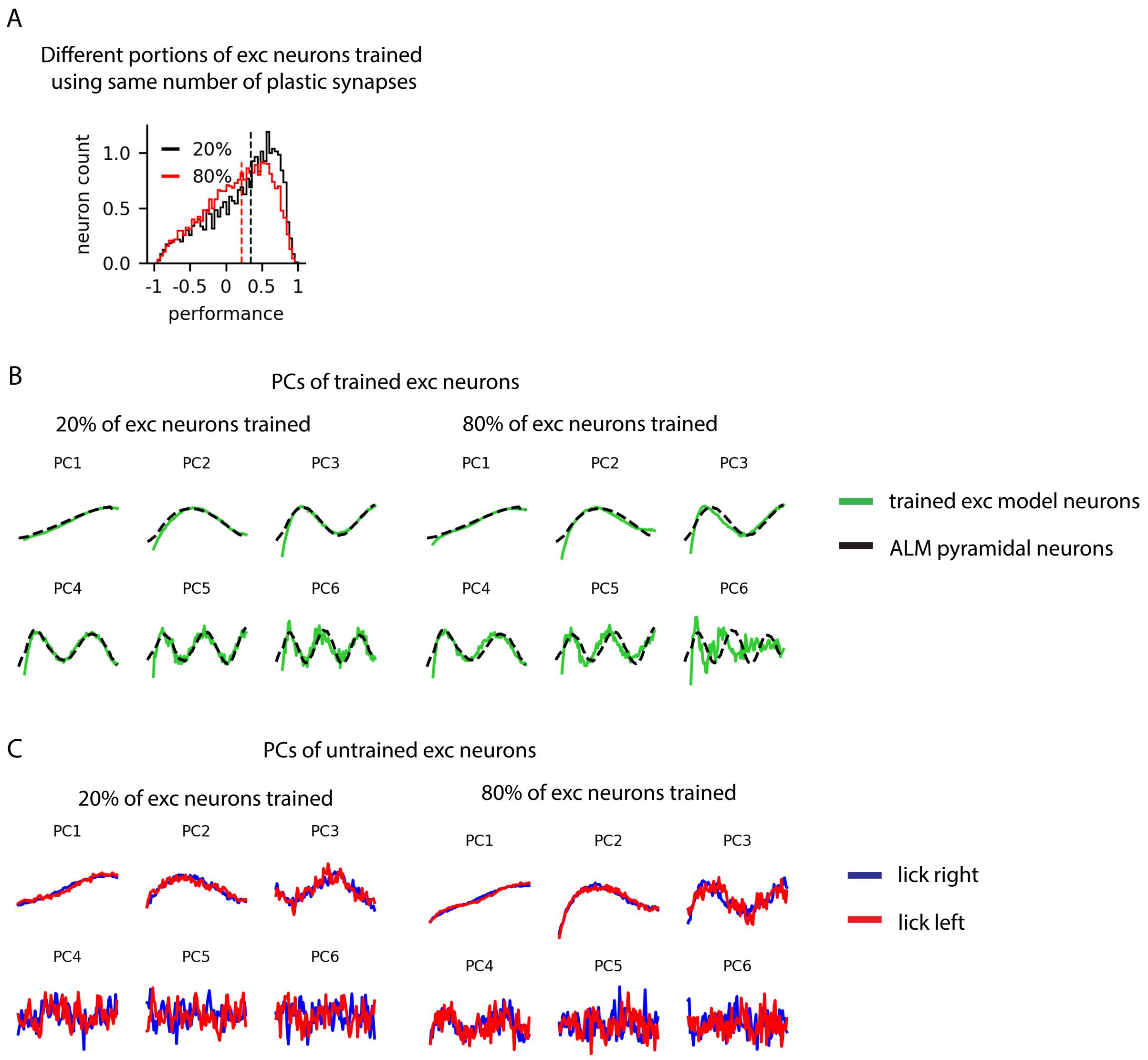
A
Different portions of exc neurons trained
using same number of plastic synapses
neuron count
1.0
0.5
0.0
20%
80%
-1 -0.5 0 0.5 1
performance
B
PCs of trained exc neurons
20% of exc neurons trained
80% of exc neurons trained
PC1 PC2 PC3
PC4 PC5 PC6
trained exc model neurons
ALM pyramidal neurons
C
PCs of untrained exc neurons
20% of exc neurons trained
80% of exc neurons trained
PC1 PC2 PC3
PC4 PC5 PC6
lick right
lick left

Fig. S11: Comparison of training 20% and 80% of excitatory neurons with a fixed total number of plastic synapses. The network consisted of $N_E = N_I = 15,000$ neurons. Each trained neuron learned to reproduce synthetic ALM pyramidal neuron activity as in Fig. 4. Here, we compared two training scenarios where 20% or 80% of excitatory neurons are trained, while keeping the total number of trainable synapses the same in both scenarios. The network with 20% of excitatory neurons trained is the one reported in Fig4A. To train 80% of excitatory neurons, we used the same total number of trainable synapses and spread the trainable synapses equally across 80% of excitatory neurons to reproduce synthetic ALM activity. **(A)** Performance of two training scenarios where either 20% or 80% of excitatory neurons were trained, using the same total number of plastic synapses. Training more neurons (80% of excitatory neurons) did not lead to improved performance. In fact, the performance was slightly decreased. This is because, as the number of trained neurons was increased, the number of trainable synapses was reduced in each trained neuron, thus lowering the learning capability. **(B)** PCs of PSTHs of trained excitatory neurons in two training scenarios, showing the responses of lick-right trial type. Differences in performance were also observed in the PCs of trained excitatory neurons. In the network with 20% excitatory neurons trained, the model PCs were in good agreement with data PCs. However, when the trainable synapses were diluted over 80% of excitatory neurons, the model PCs started to diverge from the data PCs in higher order PCs (e.g., see PC6). **(C)** PCs of PSTHs of untrained excitatory neurons in two training scenarios. In the untrained neurons, the PCs of both scenarios were mostly comparable up to PC3 and both had degraded higher mode PCs.
