# Supplementary material for: Distributing task-related neural activity across a cortical network through task-independent connections
Source: Nat Commun. 2023 May 18;14:2851. doi: 10.1038/s41467-023-38529-y (PMC10195842; doi:10.1038/s41467-023-38529-y)
Supplement: Supplementary file 3 — Reporting Summary [file 41467_2023_38529_MOESM3_ESM.pdf]

nature portfolio

Corresponding author(s): Ran Darshan, Christopher Kim,

Last updated by author(s): Feb 25, 2023

Reporting Summary

Nature Portfolio wishes to improve the reproducibility of the work that we publish. This form provides structure for consistency and transparency in reporting. For further information on Nature Portfolio policies, see our Editorial Policies and the Editorial Policy Checklist.

Statistics

For all statistical analyses, confirm that the following items are present in the figure legend, table legend, main text, or Methods section.

n/a	Confirmed	
☐	☒	The exact sample size (n) for each experimental group/condition, given as a discrete number and unit of measurement
☒	☐	A statement on whether measurements were taken from distinct samples or whether the same sample was measured repeatedly
☐	☒	The statistical test(s) used AND whether they are one- or two-sided<br>*Only common tests should be described solely by name; describe more complex techniques in the Methods section.*
☒	☐	A description of all covariates tested
☒	☐	A description of any assumptions or corrections, such as tests of normality and adjustment for multiple comparisons
☐	☒	A full description of the statistical parameters including central tendency (e.g. means) or other basic estimates (e.g. regression coefficient) AND variation (e.g. standard deviation) or associated estimates of uncertainty (e.g. confidence intervals)
☐	☒	For null hypothesis testing, the test statistic (e.g. F, t, r) with confidence intervals, effect sizes, degrees of freedom and P value noted<br>*Give P values as exact values whenever suitable.*
☒	☐	For Bayesian analysis, information on the choice of priors and Markov chain Monte Carlo settings
☒	☐	For hierarchical and complex designs, identification of the appropriate level for tests and full reporting of outcomes
☒	☐	Estimates of effect sizes (e.g. Cohen's d, Pearson's r), indicating how they were calculated

Our web collection on statistics for biologists contains articles on many of the points above.

Software and code

Policy information about availability of computer code

Data collection	Data -collection was done using:<br>SpikeGL (v 20170315) https://github.com/cculianu/SpikeGL<br>Wavesurfer (v 0.787) https://www.janelia.org/open-science/wavesurfer
Data analysis	Custom code for data analysis can be found at https://github.com/SpikingNetwork/distributedActivity

For manuscripts utilizing custom algorithms or software that are central to the research but not yet described in published literature, software must be made available to editors and reviewers. We strongly encourage code deposition in a community repository (e.g. GitHub). See the Nature Portfolio guidelines for submitting code & software for further information.

Data

Policy information about availability of data

All manuscripts must include a data availability statement. This statement should provide the following information, where applicable:

- Accession codes, unique identifiers, or web links for publicly available datasets
- A description of any restrictions on data availability
- For clinical datasets or third party data, please ensure that the statement adheres to our policy

Data in the NWB format is available for download here: https://dandiarchive.org/dandiset/000060/draft

Human research participants

Policy information about studies involving human research participants and Sex and Gender in Research.

Reporting on sex and gender	N/A
Population characteristics	N/A
Recruitment	N/A
Ethics oversight	N/A

Note that full information on the approval of the study protocol must also be provided in the manuscript.

Field-specific reporting

Please select the one below that is the best fit for your research. If you are not sure, read the appropriate sections before making your selection.

☒ Life sciences ☐ Behavioural & social sciences ☐ Ecological, evolutionary & environmental sciences

For a reference copy of the document with all sections, see nature.com/documents/nr-reporting-summary-flat.pdf

Life sciences study design

All studies must disclose on these points even when the disclosure is negative.

Sample size	The sample sizes are similar to sample sizes used in the field (Inagaki et al., Nature 2019). No statistical methods were used to determine sample size.
Data exclusions	We did not exclude any animal from data analysis.<br>We excluded sessions in which the photostimulation resulted in a significant change in<br>at least 10% of the time points during the photostimulation period. This is stated in Methods.
Replication	We performed recordings for multiple behavior sessions (n = 97 sessions) and from multiple animals (n= 9 mice) to confirm reproducibility. All presented results were reproducible across experimental replicates.
Randomization	All animals were used for experiments. Trial types were randomly determined by a computer program.
Blinding	Analysis of neural and behavior data was conducted regardless of the identity of the animal from which the data was collected. During experiments, trial types were randomly determined by a computer program. During spike sorting, experimenters cannot tell the trial type, so experimenters were blind to conditions.

Reporting for specific materials, systems and methods

We require information from authors about some types of materials, experimental systems and methods used in many studies. Here, indicate whether each material, system or method listed is relevant to your study. If you are not sure if a list item applies to your research, read the appropriate section before selecting a response.

Materials & experimental systems

n/a	Involved in the study	
☒	☐	Antibodies
☒	☐	Eukaryotic cell lines
☒	☐	Palaeontology and archaeology
☐	☒	Animals and other organisms
☒	☐	Clinical data
☒	☐	Dual use research of concern

Methods

n/a	Involved in the study	
☒	☐	ChIP-seq
☒	☐	Flow cytometry
☒	☐	MRI-based neuroimaging

Animals and other research organisms

Policy information about studies involving animals; ARRIVE guidelines recommended for reporting animal research, and Sex and Gender in Research

Laboratory animals	We used 9 adult male mice (age P>60). We used Scnn1a-TG3-Cre × Ai32 transgenic mouse line (See section 'Animals' in the Methods of Finkelstein et al. 2021 for details). Mice were maintained on a reverse 12:12-h light:dark cycle. The holding room temperature was<br>maintained at 21±1°C with a relative humidity of 30% to 70%.
Wild animals	This study did not involve wild animals
Reporting on sex	Adult male mice
Field-collected samples	This study did not involve field-collected samples.
Ethics oversight	All experimental procedures were approved by Janelia Institutional Animal Care and Use Committee.

Note that full information on the approval of the study protocol must also be provided in the manuscript.
